# MicroRNAs: At the Interface of Metabolic Pathways and Inflammatory Responses by Macrophages

**DOI:** 10.3389/fimmu.2020.01797

**Published:** 2020-08-14

**Authors:** Morgan C. Nelson, Ryan M. O'Connell

**Affiliations:** ^1^Department of Pathology, Division of Microbiology and Immunology, University of Utah, Salt Lake City, UT, United States; ^2^Huntsman Cancer Institute, University of Utah, Salt Lake City, UT, United States

**Keywords:** microRNA, metabolism, macrophage, immunometabolism, inflammation, macrophage polarization

## Abstract

Macrophages are key cells of the innate immune system with functional roles in both homeostatic maintenance of self-tissues and inflammatory responses to external stimuli, including infectious agents. Recent advances in metabolic research have revealed that macrophage functions rely upon coordinated metabolic programs to regulate gene expression, inflammation, and other important cellular processes. Polarized macrophages adjust their use of nutrients such as glucose and amino acids to meet their changing metabolic needs, and this in turn supports the functions of the activated macrophage. Metabolic and inflammatory processes have been widely studied, and a crucial role for their regulation at the post-transcriptional level by microRNAs (miRNAs) has been identified. miRNAs govern many facets of macrophage biology, including direct targeting of metabolic regulators and inflammatory pathways. This review will integrate emerging data that support an interplay between miRNAs and metabolism during macrophage inflammatory responses, highlighting critical miRNAs and miRNA families. Additionally, we will address the implications of these networks for human disease and discuss emerging areas of research in this field.

## Introduction

The immune system consists of a vast array of innate and adaptive immune cells necessary for defense against pathogen invasion. Macrophages are key constituents of the innate immune system and are an integral part of the first response to infection through their recognition of pathogen-associated molecular patterns (PAMPs) by pattern recognition receptors (PRRs) that include toll-like receptors (TLRs), and NOD-like receptors (NLRs), among others (1–3). Following detection of microbes, macrophages take on a number of roles such as directly killing invading pathogens, phagocytizing dead and dying cells, responding to parasite invasion through cytokine release, and priming the adaptive immune system to generate antigen-specific responses against infectious agents (4, 5).

Macrophages are widely distributed throughout the body, residing in nearly every tissue (6). These tissue resident macrophages emerge from distinct sources—either originating from the prenatal yolk sac and seeding into tissues during embryonic development (7), or arising from the bone marrow as monocytes and circulating through the bloodstream prior to injury or infection, prompting differentiation and tissue infiltration (8). Tissue macrophages respond to cytokines, microbial signals, fatty acids, or growth factors in their environment (8), which leads to changes in transcription, cell surface protein expression, and cellular function. Considerable heterogeneity exists among activated macrophage populations (6, 9), and these phenotypic changes are generally described as one of two main polarization subtypes: classical (“M1”) and alternative (“M2”). M1-polarized macrophages are typically activated by inflammatory cytokines such as interferon gamma (IFN-γ) and TLR ligands such as lipopolysaccharide (LPS), and promote pathogen clearance through inflammation and microbicidal activity (10). M2-polarized macrophages, on the other hand, usually respond to Interleukin-4 (IL-4) and IL-13 and promote parasite clearance, inflammatory resolution, and tissue repair (11). The M1/M2 classification system provides a simple paradigm to describe macrophage phenotypes based on their more prominent defining features during activation, yet it simplifies the plastic nature of the macrophage response spectrum, which is subject to an array of activating signals, and which has been extensively discussed in other reviews (12, 13). This review provides a list of relevant activating signals in Table 1 to provide a more in-depth perspective on macrophage activation and polarization.

**Table 1:** Functional roles of microRNAs in metabolism, inflammation, and disease.

**miRNA**	**Relevant targets**	**Induced by**	**Role in inflammation or metabolism**	**Role in disease outcome**	**References**
miR-155	SOCS1, SHIP1	LPS/NF-kB	Promotes M1 inflammatory response	Promotes myeloproliferative disease	(14)
miR-146a	TRAF6, IRAK1	LPS/NF-kb	Inhibits M1 inflammation, inhibits glycolysis through mTOR signaling	Protects against obesity; Promotes Sjögren's	(15, 16)
miR-21	PTEN, PDCD4, PFK-M	NF-kB or AP-1	Inhibits M1 inflammation, promotes IL-10 production, inhibits glycolysis	Promotes cancer, promotes Mycobacterium tuberculosis infection	(17, 18)
Let-7adf	A20, LIN28a, TET2	LPS	Promotes M1 inflammation, promotes metabolic activation and IL-6 production	Reduces Mycobacterium tuberculosis and Salmonella infections	(19, 20)
Let-7b	?(unknown in macrophages)	Cancer cell conditioned media	Regulates inflammatory cytokines IL-12, Il-23, TNF-a; promotes TAM phenotype	Promotes carcinoma	(21, 22)
Let-7c	C/EBP-δ, PAK1	Suppressed by LPS	Promotes M2 polarization, depletes CCR7, and MHC-II levels	Implicated in promoting pulmonary fibrosis	(23, 24)
Let-7e	TLR4, IRAK1	AKT, LPS	Suppresses M1 polarization	Promotes endotoxin tolerance	(25, 26)
miR-99a	TNF-a	IL-4	Promotes M2 phenotype and reduces, inflammatory cytokines	Reduces adipose tissue inflammation and protects from diabetes	(27)
miR-34	NOTCH1	Suppressed by LPS	Inhibits M1 inflammation	Protects from diet-induced obesity	(28, 29)
miR-30	DLL4, REDD1	Suppressed by HIF1α	Inhibits M1 inflammation, regulates glycolytic capacity	Downregulated during obesity; protects against gastric cancer	(30, 31)
miR-125a	A20, FIH1, IRF4, KLF13	LPS/NF-kB, NOTCH1	Promotes or inhibits M1 inflammation, promotes HIF1α, increases phagocytosis	Protects against cancer; complex roles in inflammation	(32, 33)
miR-125b	BIK, MTP18	IFN-γ + LPS	Promotes M1 inflammatory response	Protects against chronic inflammatory systemic disorder	(34)
miR-33	ABCA1, CPT1a, AMPK	IFN-γ + LPS	Inhibits fatty acid oxidation and promotes glycolysis, inhibits M2 phenotype	Promotes atherosclerosis	(35)
miR-150	SCD2	LPS, LDL	Regulates lipid traffickiing and promotes angiogenesis	Promotes macular degeneration	(36)
miR-17/20a	HIF1α and HIF2α	Repressed by PMA	Regulate macrophage differentiation, repress hypoxic activity of HIF proteins	Implicated in inhibiting angiogenesis in tumors	(37, 38)
miR-210	NF-kB, NDUFA4	HIF1α, LPS	“hypoxamir”; regulates metabolism, inflammation, and cell proliferation	Promotes parasite infection; promotes diabetes	(39–41)
miR-511	ROCK2	IL-4	Transcribed with CD206, promotes inflammation	Limits pro-tumoral functions in TAMs; Promotes colitis	(42, 43)
miR-221/222	Brg1	LPS	Involved in macrophage tolerance	Promotes sepsis	(44)

Macrophages are important for normal homeostatic maintenance and contribute to self-limited inflammation during infection; however, these beneficial functions can be superseded by prolonged activation signals leading to dysregulated macrophage activity, and this can have pathological consequences (5). For instance, unrestrained or chronic inflammation from macrophages can drive inflammatory conditions such as metabolic disease, cytokine storm, and sepsis (45–47). On the other hand, macrophages sometimes fail to mount a sufficient inflammatory response, especially within tumors where they are referred to as tumor-associated macrophages (TAMs). TAMs often resemble M2 macrophages and promote angiogenesis, decrease antigen presentation, dampen inflammatory cytokine production, and impede T cell recruitment into the tumor (48). Research in this field has uncovered many mechanisms that have evolved to regulate macrophage activity and aid in controlling the inflammatory response.

In recent years, the field of immunometabolism has expanded rapidly. It is now widely accepted that leukocyte metabolism is an essential part of a coordinated immune response. Macrophage metabolism in particular has been widely studied, and it has become clear that metabolism plays a considerably larger role in macrophages than simply driving energy production for the cell. Rather, it is a dynamic process with direct and context-specific roles in driving inflammatory signaling and other macrophage effector functions (49, 50). It has also become evident that metabolic programs are responsive to many cues beyond nutrient availability, including cytokine-mediated polarization signals (51, 52). Under polarizing conditions, M1 and M2 macrophages differentially utilize metabolic pathways to promote their inflammatory or anti-inflammatory functions. For instance, M1 macrophages rely on aerobic glycolysis for their energetic needs, and this polarized metabolic programming drives the pentose phosphate pathway (PPP), itaconate production, and nitric oxide (NO) synthesis, all of which support the functions of classically activated macrophages. Conversely, M2 macrophages use oxidative phosphorylation (OXPHOS) for their energetic needs, driving glycosylation of cell surface receptors and producing ornithine to support wound healing functions of alternatively activated macrophages (52). The metabolic activity of macrophages has been an exciting area of research in recent years and has greatly expanded our understanding of how macrophage responses are regulated.

Inflammatory and metabolic gene expression must be tightly controlled during macrophage activation for a proper macrophage response to occur. Cellular mechanisms exist to ensure this, with one of these being post-transcriptional gene regulation by miRNAs. miRNAs are small, ~22 nucleotide non-coding RNAs that are fundamental in coordinating expression of a diverse array of genes in immune populations (53). These small RNAs are endogenously expressed and are generated first as long primary transcripts which undergo a series of processing steps to reach their final mature state (54). Once formed, the mature miRNA is loaded into the RISC complex and acts as a guide to complementary mRNA targets. The miRNA-RISC complex binds to the target mRNA, leading to either degradation or translational inhibition of the mRNA transcript. miRNAs are best known for acting intracellularly, but they can also be loaded into exosomes for transport to distant cells (55). A single miRNA can target multiple genes; likewise, a single gene can be repressed by numerous miRNAs, and it is thought that at least 60% of all protein-coding genes are under miRNA regulation (56). miRNAs are numbered sequentially by discovery date and classified into families based on sequence homology, structure, or functional similarities (57).

These small but critically important molecules have been shown to regulate gene expression during each stage of macrophage development, from myelopoiesis all the way through polarization and during effector function (58), and have been identified as a regulatory link between polarization signals and metabolic function (59). miRNAs thus ensure the ability to properly gauge key macrophage processes and inflammatory programs.

miRNAs have distinct roles in regulating macrophage activities to restrain macrophage-associated diseases; meanwhile, miRNA dysregulation is associated with poor prognosis in many of these diseases. Recent work in this field has identified specific miRNAs and their mechanistic functions in macrophages, playing a part in both health and disease. In this review, we will discuss macrophage metabolic and inflammatory programming and the important functional consequences of miRNAs in controlling these processes. As we examine the existing research in this field of study, it should be mentioned that some differences exist between human and mouse macrophages, including distinct metabolic and miRNA variations (60); however, these molecules and processes are also conserved in many cases.

## Metabolic Programming in Polarized Macrophages Is Essential for Function

Macrophage activation relies heavily on the metabolic activities of the cell to fuel a specific and timely response. Polarized macrophages rely on specific metabolic pathways to promote the dynamic activities of pro- or anti-inflammatory responses, and metabolic gene expression levels are often used as markers of classically or alternatively activated macrophage populations (13). In this section we will discuss the metabolic changes that occur in macrophages during polarization to promote macrophage functions.

Prior to polarization, macrophages exhibit a resting metabolic state that relies on OXPHOS fueled by the tricarboxylic acid (TCA) cycle for energy needs. When classical activation is initiated by extracellular molecules such as IFN-γ and LPS, murine macrophages undergo a drastic increase in energy demand and induce heightened glucose flux to meet these energetic needs. Macrophage polarizing signals initiate master transcription factors such as nuclear factor-κB (NF-κB) and hypoxia-inducible factor 1α (HIF1α), among others, to carry out changes in inflammatory gene expression and metabolic programming (61, 62). During polarization, inflammatory macrophages switch their metabolic programming to favor aerobic glycolysis over OXPHOS (51, 63, 64). Although aerobic glycolysis, also termed the Warburg Effect, is less efficient at generating ATP than is OXPHOS, the heightened glucose flux into the cell allows for rapid energy production and is used to generate other biosynthetic intermediates as well (65). This includes the pentose phosphate pathway, which supplies NADPH for reactive oxygen species (ROS) and NO production, and which helps maintain the cellular redox state of the cell (66). The pentose phosphate pathway also generates intermediates necessary for nucleotide synthesis, although the need for nucleotide production is unclear since activated macrophages do not proliferate (67). Finally, aerobic glycolysis results in pyruvate being reduced to lactate, which maintains the glycolytic flux in activated macrophages (68).

A hallmark of inflammatory macrophage metabolism is a defective tricarboxylic acid (TCA) cycle. The TCA cycle, which is primarily fed by the products of glycolysis and glutamine metabolism, is broken in two spots in inflammatory murine macrophages, resulting in a buildup of TCA intermediates (64, 69, 70). These breakpoints occur at isocitrate dehydrogenase (IDH), leading to citrate accumulation, and at succinate dehydrogenase (SDH), leading to succinate accumulation (64, 71, 72). These accumulated metabolites are then used for other metabolic and immune signaling functions. First, accumulated citrate is used to drive fatty acid synthesis and NO production to support a classically activated macrophage phenotype (68). Citrate can also be converted into the antimicrobial metabolite itaconate (73, 74), which has multiple roles, including antimicrobial protection against intracellular pathogens and inhibition of SDH to promote succinate buildup (75). Second, accumulated succinate stabilizes HIF1α and leads to sustained IL-1β and ROS production (64). IL-1β is among the inflammatory cytokines characteristically produced by IFN-γ-induced macrophages, along with TNF-α, IL-6, and IL-12 (12). HIF1α is a key transcription factor in regulating both the glycolytic and inflammatory capacities in macrophages, and its absence results in impaired motility, defective bactericidal activity, and poor macrophage accumulation (76). The function of itaconate and succinate in driving antimicrobial programs and pro-inflammatory cytokine expression, respectively, are prime examples of the direct role of metabolic products on macrophage inflammatory function.

This central role for metabolism is further illustrated by seminal findings in the early 1990s demonstrating that murine macrophage subtypes differentially metabolize the amino acid arginine (77–79). These studies showed that inflammatory polarization induces expression of the enzyme NOS2, which converts arginine to the microbicidal molecule NO. NO is cytotoxic to pathogens and thus aids in the antimicrobial macrophage response. In contrast, wound healing macrophages hydrolyze arginine to the tissue-remodeling amino acid ornithine through expression of the enzyme ARG1. Ornithine promotes wound healing by aiding in collagen formation and inhibiting inflammatory cytokine expression (80–83). These distinct metabolic activities in pro- or anti-inflammatory macrophages are driven by macrophage polarization signals, and in return the products of arginine metabolism promote the specific functions of polarized macrophages.

**Figure 1:**
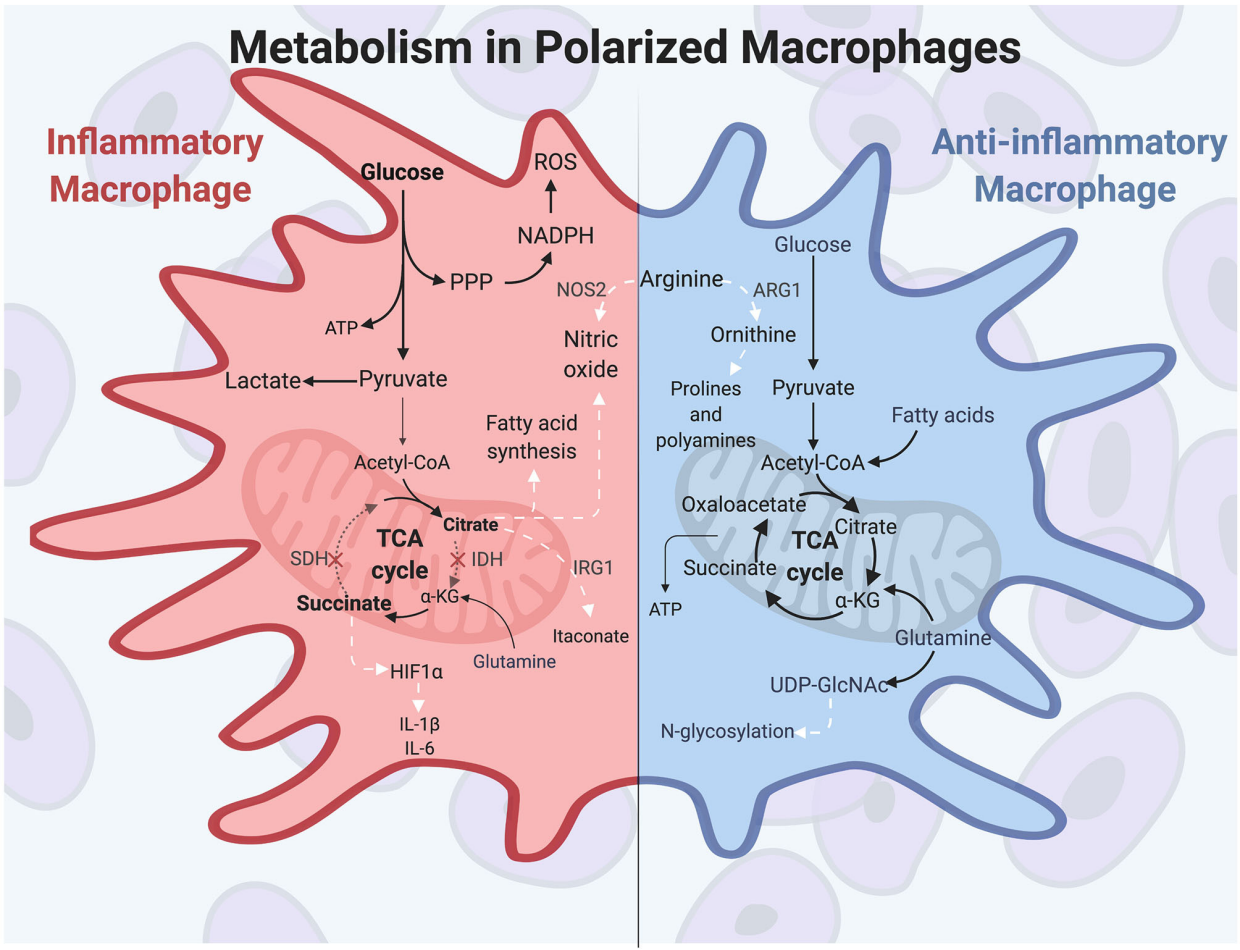
Polarized macrophages have differential metabolic programming. *Inflammatory macrophages* characteristically increase glucose uptake to fuel aerobic glycolysis, producing increased lactate and ATP production. The pentose phosphate pathway feeds off of the increased flux of glycolytic intermediates, resulting in heightened nucleotide and amino acid synthesis. Classically activated macrophages have a disrupted TCA cycle in two locations: at isocitrate dehydrogenase (IDH) and at succinate dehydrogenase (SDH). As a result, citrate and succinate accumulate and drive such functions as fatty acid and NO synthesis, antimicrobial itaconate production, and HIF1α activity to promote further glycolysis and inflammatory cytokine production. Arginine is differentially metabolized in polarized murine macrophages, producing the antimicrobial molecule NO in inflammatory macrophages. *Anti-inflammatory macrophages* are not as metabolically active compared to classically activated macrophages, and they utilize glutamine, glucose, and fatty acids to fuel the TCA cycle and OXPHOS. In alternatively activated macrophages, glutamine uptake drives UDP-GlcNAc production, which is important for N-glycosylation of cell surface receptors, such as CD206 and CD301. In anti-inflammatory murine macrophages, arginine is metabolized to ornithine, which promotes tissue repair through production of prolines and polyamines. White arrows indicate metabolic intermediates driving M1 or M2 activities. α-KG, alpha-ketoglutarate; ARG1, arginase; HIF1α, hypoxia-inducible factor 1α; IDH, isocitrate dehydrogenase; IRG1, immune-responsive gene 1; NOS2, nitric oxide synthase; PPP, pentose phosphate pathway; ROS, reactive oxygen species; SDH, succinate dehydrogenase.

While the metabolic profile undergoes dramatic changes upon pro-inflammatory polarization, the anti-inflammatory macrophage triggered by IL-4 and IL-13 more closely resembles metabolism seen in non-polarized macrophages. Alternatively activated murine macrophages use glucose and glutamine to feed the TCA cycle, and also rely on fatty acid oxidation (FAO) to fuel OXPHOS (84). Important transcription factors and enzymes are involved in alternative macrophage activation and are distinct from inflammatory macrophages. Among these transcription factors, IRF4 is responsive to IL-4 stimulation and supports the roles of inflammatory abatement, wound repair, and angiogenesis, and it contributes to metabolic reprogramming in alternatively activated macrophages (85). Additional molecules are important in M2 macrophages, such as the kinase AMP-activated protein kinase (AMPK), which is induced in response to anti-inflammatory cytokines in murine macrophages and plays a role in stimulating fatty acid oxidation (FAO) (84, 86). Further, the coenzyme UDP-GlcNAc, a product of glucose and glutamine metabolism, is a metabolite produced during alternative macrophage activation which glycosylates receptors, such as the mannose receptors CD206 and CD301. The loss of this necessary enzyme diminishes alternative polarization and cell surface receptor expression (11, 13, 74, 87). Finally, anti-inflammatory macrophages characteristically produce cytokines such as IL-10 and TGF-β, and these cytokines promote alternative activation through multiple mechanisms, including repressing metabolic remodeling in inflammatory macrophages (88). Overall, metabolic programs in polarized macrophages are distinct from one another but promote the inflammatory or reparative functions of the specific macrophage responses. These functions are summarized in Figure 1.

## MiRNA-Mediated Regulation of Macrophage Function

**Figure 2:**
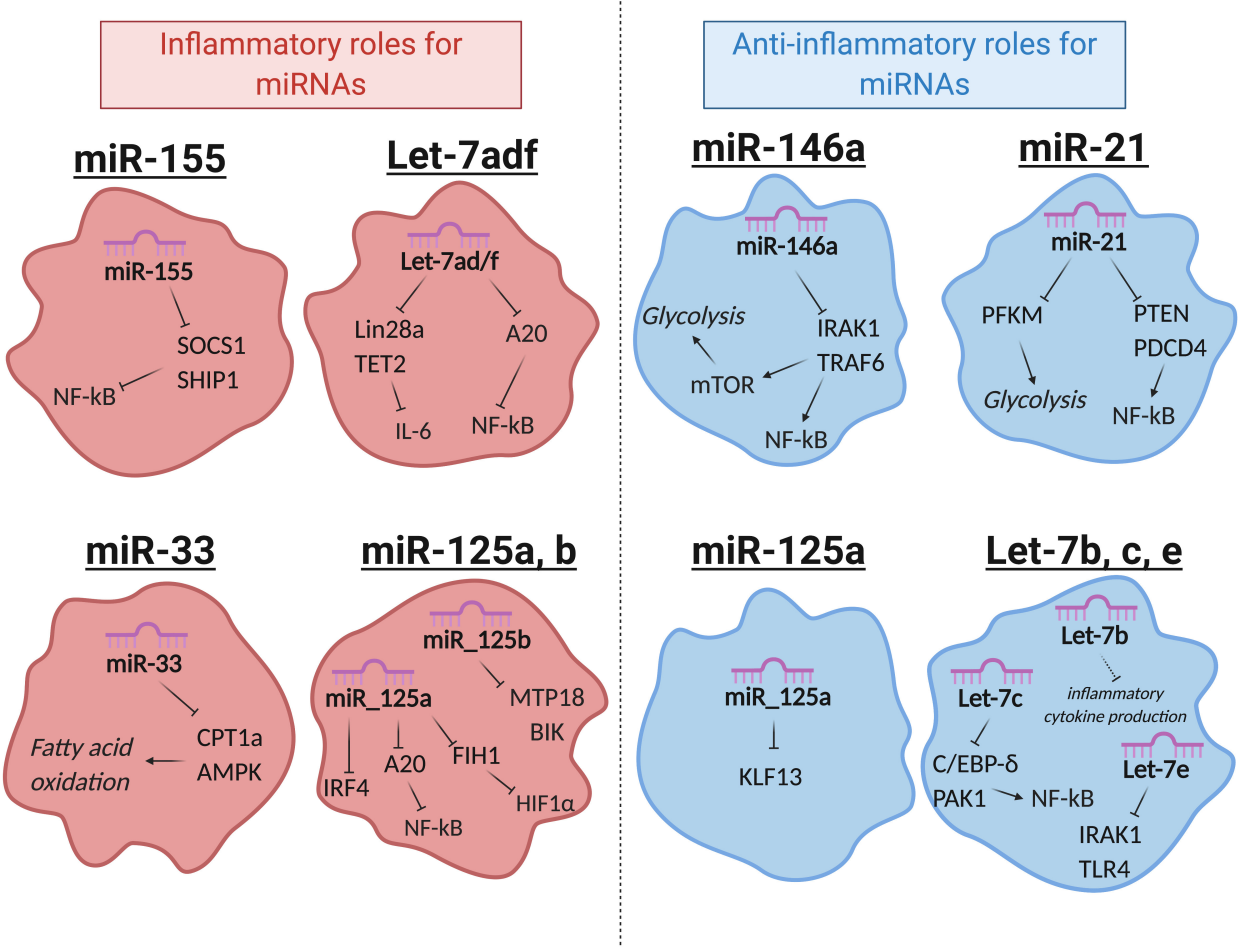
Selected miRNAs that regulate macrophage metabolic and inflammatory pathways. miRNAs are involved in regulating a number of important processes in macrophages. Certain miRNAs, highlighted here, target inflammatory or metabolic pathways to regulate macrophage activities. Red (left): miRNAs that promote inflammatory polarization. Blue (right): miRNAs that promote anti-inflammatory polarization. MiR-125a has noted roles in promoting both inflammatory and anti-inflammatory macrophages, as indicated. Let-7e is part of the miR-99b~Let-7e~miR-125a cluster and acts coordinately with miRNAs in its cluster to have the functions shown.

miRNAs that regulate distinct aspects of macrophage responses have been identified, including those that either promote or mute inflammation in response to polarizing signals. More recently, a growing body of literature has emerged demonstrating the role for miRNAs in regulating metabolic functions to impact macrophage responses (89). While regulation of innate immune metabolism by miRNAs is still up-and-coming, the literature available in this area suggests a strong link between miRNA function and metabolic proficiency in macrophages, with aberrant miRNA expression being linked to deficient antimicrobial responses, metabolic syndromes, and cancer. Here we will review miRNAs that regulate inflammation or metabolism, miRNAs that have been identified to regulate both, and we will discuss how these miRNAs impact macrophage function (Figure 2 and Table 1).

### miR-155

miR-155 was one of the first miRNAs found to regulate the immune system and is one of the most widely studied miRNAs in immunology. It is expressed in both lymphoid and myeloid lineages and is highly induced in macrophages in response to the key inflammatory transcription factor NF-κB (90–93). miR-155 promotes the inflammatory response in humans and mice by targeting SOCS1 and SHIP1, which are negative regulators of inflammation. miR-155 promotes inflammatory cytokine production, PI3K/AKT activity, type I IFN signaling, and further NF-κB function through silencing its anti-inflammatory targets (25, 94–97), and loss of miR-155 attenuates the macrophage antiviral response, highlighting its role in driving inflammatory macrophage function (98, 99).

### miR-146a

In contrast to miR-155, miR-146a represses the inflammatory response by targeting transcripts of TRAF6 and IRAK1, two proteins that promote NF-κB activity in the TLR signaling pathway (100). Overexpression of miR-146a diminishes inflammatory cytokine production, while deletion or inhibition of miR-146a leads to elevated inflammatory markers in both human and mouse macrophages, illustrating its role in negatively regulating inflammation (101–103). Recently, our group identified an additional function for miR-146a, showing that it has the ability to regulate metabolic pathways in murine macrophages (15). We found that miR-146a does this by impeding mTOR signaling through its target, Traf6. This restrains glycolysis; further, genetic knockout of miR-146a leads to highly glycolytic and hyperinflammatory macrophages and gives rise to diet-induced inflammation and obesity in mice. These findings demonstrate a dual role for miR-146a in promoting both metabolic and inflammatory abatement.

### miR-21

miR-21 is another miRNA exhibiting synchronous roles in both inflammation and metabolism. miR-21 is an endotoxin-responsive miRNA, shown to be induced by either NF-κB or AP-1 (104, 105). This miRNA promotes an anti-inflammatory macrophage phenotype and inflammatory resolution in mouse and human cells by targeting transcripts of the inflammatory proteins PTEN and PDCD4. This leads to decreased NF-κB signaling and increased anti-inflammatory IL-10 production (106, 107). Consistent with this, miR-21 expression in macrophages promotes TAMs which support tumor growth, and its suppression leads to an improved anti-tumor response (17). In addition to targeting inflammatory genes, miR-21 also targets the glycolytic pathway enzyme PFK-M for downregulation (18). This enzyme catalyzes an early step in glycolysis, and its inhibition negatively regulates glycolytic flux (108). Hackett et al. found that miR-21 induction by *Mycobacterium tuberculosis* during infection limits the switch to a glycolytic state, thus inhibiting inflammatory macrophage polarization. *M. tuberculosis* is known to promote its own survival by inducing host anti-inflammatory or anti-glycolytic factors (109), and thus sustained miR-21 expression in macrophages is tolerogenic to bacterial growth. miR-21 exhibits roles in restraining pro-inflammatory macrophage activation by targeting both inflammatory and metabolic factors.

### Let-7

The Let-7 family of miRNAs contains 10 mature miRNAs in humans, many of which are relevant in macrophage metabolism and have functions in the immune response to pathogens (21, 23, 110). Let-7 was one of the first discovered miRNAs, originally found in *C. elegans* but with high conservation across species (53). These miRNAs have been extensively studied and a number of them will be highlighted here. First, Let-7f promotes inflammatory polarization and cytokine production in murine macrophages by targeting the negative regulator of NF-κB, A20. During *M. tuberculosis* infection, Let-7f expression is downregulated and A20 levels rise, leading to diminished NF-κB activity and a poor inflammatory macrophage response (19). This study further showed that Let-7f is sufficient to increase inflammatory NF-κB expression in macrophages and concordantly regulate bacterial growth, as *M. tuberculosis* survival was diminished after transfection of Let-7f mimics.

Let-7f can also regulate immune metabolism. Let-7f is part of the let-7a-1/let-7d/let-7f-1 (let7-adf) cluster of coordinately transcribed miRNAs, which has been extensively studied for its role in regulating metabolism in multiple immune populations (20, 111). In LPS-activated macrophages, Let-7adf stimulates proinflammatory IL-6 cytokine expression and promotes inflammation through metabolic targets. It does this by targeting two genes in the succinate pathway (20). First, let-7adf regulates one of its well-established targets LIN28a, which directly binds to and enhances SDH activity. By diminishing LIN28a expression, the let-7adf cluster dampens SDH activity and promotes succinate accumulation. Second, let-7adf also inhibits TET2, which normally acts to repress IL-6 downstream of succinate. Inhibition of these two genes by let-7adf promotes succinate accumulation and IL-6 expression.

While Let-7f promotes inflammatory macrophage activity, a number of Let-7 family members instead promote inflammatory resolution. Among these are Let-7b, which promotes anti-inflammatory phenotypes by regulating the inflammatory cytokines IL-12, IL-23, and TNF-α and stimulating angiogenesis in human TAMs (21). Let-7c, another Let-7 family member, promotes wound healing phenotypes by targeting and repressing C/EBP-δ and PAK1, a transcription factor and protein kinase, resulting in reduced NF-κB and AP-1 activity (23, 24). Let-7c overexpression leads to diminished proinflammatory cytokine expression, depleted CCR7 levels, and attenuated MHC-II surface expression in murine macrophages, all indicative of M2 polarization. Finally, let-7e, which falls within the coordinately transcribed let-7e~miR-99b~miR-125a cluster of miRNAs, acts with its cluster members miR-99b and miR-125a to target a number of genes to suppress inflammatory polarization, including TLR4 and IRAK1 (25, 26, 112).

### miR-125

As mentioned above, miR-125a is part of the let-7e~miR-99b~miR-125a cluster of miRNAs, though this miRNA may have complex and independent roles from the rest of its cluster in macrophage metabolism and activation. miR-125a promotes pro-inflammatory macrophage responses by targeting FIH1, a negative regulator of HIF1α, and by suppressing the transcription factor IRF4 (32). Additionally, miR-125a has been shown to target A20 to promote NF-kB function (33). In contrast, miR-125a has been reported to have anti-inflammatory roles in macrophages by targeting KLF13, a transcription factor important for inflammation and T lymphocyte activation (113). In this latter context, miR-125a initiation occurs late relative to LPS stimulation. While it has not been shown experimentally, the let-7e~miR-99b~miR-125a cluster may rely on transcriptional timing or relative abundance of distinct miRNAs within this cluster to dictate whether it will favor inflammation or inflammatory resolution since miRNAs in this cluster seem to have opposing roles (114). miR-125a has complex functions in macrophage polarization, but the cluster as a whole has been shown to favor an anti-inflammatory phenotype (26), leading to the idea that cellular context and abundance of individual miRNAs could promote different macrophage programs.

miR-125b is part of the miR-125 family, and it has functions in both inflammation and metabolism to promote an inflammatory response in human and mouse macrophages (34, 115). IFN-γ and LPS stimulation induce miR-125b, which in turn regulates the anti-inflammatory transcription factor IRF4 (115). Further, this miRNA targets genes associated with OXPHOS to promote glycolysis in human monocytes. miR-125b targets BIK, a proapoptotic protein that promotes oxygen consumption, and MTP18, a mitochondria-localized protein involved in mitochondrial fission (34, 116). By targeting these genes, miR-125b abrogates the oxidative capacity of the mitochondria and supports pro-inflammatory activation.

### miR-33

A number of publications have focused on the functions of miR-33a and miR-33b, which are co-transcribed with their host genes, SREBF1 and SREBF2 (117–119). These host genes are cholesterol and fatty acid-regulating transcription factors, and studies revealed that miR-33a and b promote the functions of their host genes by targeting genes such as ABCA1 to dampen cholesterol efflux and CPT1a to regulate FAO. Furthermore, it was shown in mouse macrophages that miR-33 targets AMPK, which is a protein kinase important for FAO. miR-33 dampens FAO and promotes glycolytic programming, and thus drives inflammatory polarization (35). miR-33 deletion in macrophages results in M2 polarization that is atheroprotective in hypercholesterolemic mice, demonstrating a physiological role for the activity of this miRNA in a disease driven by inflammatory macrophages.

### Additional miRNAs

While this list highlights many crucial miRNAs that regulate macrophage immune responses, a number of other miRNAs have important roles in macrophage function as well. Additional miRNAs are known to dampen inflammation, such as miR-99a, which targets TNF-α (27), and miR-34 and miR-30, which regulate NOTCH signaling to restrain the inflammatory response (28, 30). Conversely, additional miRNAs are known to promote inflammation, such as miR-27a which targets IL-10 to support an inflammatory immune response (120). Other miRNAs have also been noted for their relevance in regulating different aspects of macrophage metabolism. Among these, miR-150 targets SCD2 to regulate lipid metabolism and angiogenesis, promoting macular degeneration in mice (36). miR-17 and miR-20a, which are transcribed as part of the miR-17-92 cluster, regulate HIF1α and HIF2α expression, playing a role in macrophage gene regulation in differentiating monocytes and in TAMs (37, 38). It has become clear from the research in this field that miRNAs are integral for regulating important metabolic processes in macrophages and that their activities promote immune responses. As this field continues to expand, more miRNAs will likely be identified with crucial metabolic roles, and these findings will help us to better understand and ultimately treat diseases.

## Looking Ahead: Could Metabolism Play a Role in Regulating miRNAs?

**Figure 3:**
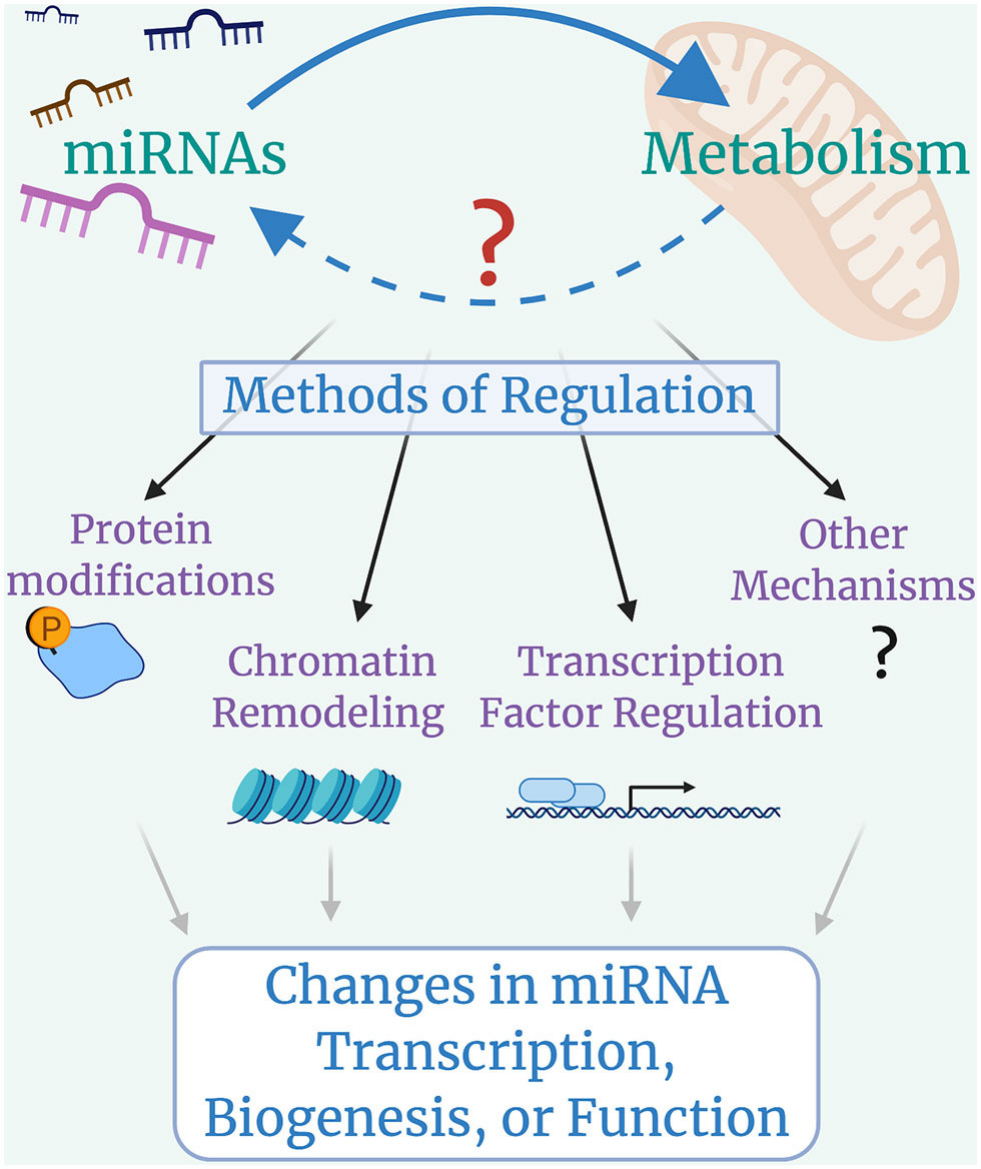
Looking forward: cross-talk between microRNAs and Metabolism. miRNAs control metabolic processes, but it is still largely unclear whether metabolic programs regulate miRNA transcription, biogenesis, or function. Metabolically-induced mechanisms of miRNA regulation could include modifications to miRNA machinery proteins such as DROSHA, DICER, or AGO2, with global impacts on miRNA biogenesis; histone modifications that lead to chromatin remodeling and alterations in miRNA availability; changes in transcription factor activity that lead to up-regulation or down-regulation of specific miRNAs; and other possible mechanisms that have yet to be identified.

With the recent identification of miRNAs regulating metabolic pathways, it is possible that cross-talk exists between miRNAs and metabolism (Figure 3). Identifying whether metabolism can broadly impact miRNA transcription, biogenesis, or function is a natural next step for this field, owing to the fact that metabolic regulation of protein-coding gene expression and cellular function has been a thriving area of research for many years now.

The best current examples of metabolically controlled miRNAs are those regulated under hypoxic conditions by the transcription factor HIF1α. A handful of these miRNAs, termed “hypoxamirs,” have been identified, with miR-210 being one of the best-studied. This miRNA is induced by HIF1α in both hypoxic and normoxic conditions, and it in turn regulates metabolism, inflammation, and cell proliferation in macrophages (39, 121–124). Another miRNA, miR-30c, has also been shown to act under HIF1α control to regulate macrophage glycolytic capacity in human macrophages (31). It is important to note that while these examples are of metabolically-regulated miRNAs in macrophages, others have been identified in different cell types as well (125).

Another promising area is in cancer metabolism. Altered metabolism is a hallmark of cancer and it is well-established that miRNAs are dysregulated during cancer, both in immune and non-immune populations (126, 127). Altered metabolic pathways in macrophages impact their function in tumor microenvironments (128, 129), and it is easy to speculate that these metabolic changes could impact miRNAs as part of their regulatory networks. As a result, cancer could be a phenomenal disease model to study how metabolic pathways affect miRNAs to induce differential disease outcomes.

## Discussion

In summary, macrophages play a distinct role in maintaining immune system homeostasis and initiating inflammatory responses. We have described how metabolism is an integral part of the macrophage response and how miRNAs are critical mediators of gene expression to either enact or abate inflammatory signals. It is evident that metabolism contributes to the macrophage effector function both by providing needed energy and also through extra-metabolic functions of metabolites to regulate the inflammatory process. miRNAs evidently control both inflammation and metabolism to coordinate a macrophage response. Some of the key miRNAs that control macrophage inflammation and metabolism which we highlighted here include Let-7f, which promotes inflammation, and miR-146a and miR-21, which promote inflammatory resolution. It is certainly possible that additional crucial miRNAs have yet to be described. The fine-tuned effects of miRNAs in macrophages protect the host against disease, and miRNA dysregulation can lead to macrophage-related pathologies.

Despite a robust understanding of the role of miRNAs in macrophage activation and metabolic programming, there are still many open questions. One of the most intriguing new frontiers to address in coming years will be the mechanisms regulating cross-talk between metabolism and miRNAs as we discussed in this review. This question relates not only to specific miRNAs, but can also be applied to global miRNA biogenesis being affected by metabolism (130), which has started to be explored. It was recently shown that miRNA biogenesis processing proteins are regulated in a metabolism-dependent manner, albeit in non-macrophage cells. Drosha, the nuclear protein that processes miRNAs, can be modulated in response to glucose availability, and this has a profound effect on global miRNA biogenesis (131). In addition to this work, other studies have shown that global miRNA regulation greatly affects macrophage function (132–135). Whether and to what extent metabolism regulates miRNA biogenesis in macrophages is a very compelling concept for future study. This will surely be a frontier in immunometabolic research as this exciting area of study moves forward.

Second, miRNAs as extracellular messengers by exosomes-mediated transport is also an intriguing area of research. The full extent of this communication network is still unclear, but some work has revealed that exosomal miRNAs play functional roles on recipient tissues in an endocrine manner and can regulate metabolic processes such as insulin signaling (136). It is still largely unclear what effect macrophage exosomes have on recipient cells and whether or how metabolic functions play a role in exosome biology.

Finally, whether species differences between human and mouse macrophages, including metabolic, inflammatory, and miRNA differences, impact their overall function and phenotype has been a longstanding topic of debate (60). For example, human macrophages induced by LPS do not express NOS2 during arginine metabolism, while NOS2 is a hallmark gene of LPS activation in murine macrophages (60, 137). Limitations in human sampling and variations in commonly used cell sources (for instance, mouse bone marrow-derived macrophages, human peripheral blood macrophages, and mouse or human immortalized cell lines) could contribute to noted phenotypic differences between species. Improving sampling and standardizing methodology for studying macrophage polarization could help improve our understanding of pre-clinical mouse models for human disease research and drug development.

As this field continues to grow, the potential for therapeutic interventions will continue to expand. Clinical trials have shown promise for targeting miRNAs for inhibition (138, 139), and others are exploring miRNA mimics for use in miRNA replacement therapy (140, 141). However, there are still many hurdles to overcome regarding developing and delivering miRNA therapies, and a deeper understanding of miRNA physiology will certainly lend itself to better therapeutic strategies and thus improved clinical outcomes. Furthermore, targeting macrophage metabolism for therapeutic purposes is emerging with the potential to significantly improve cancer, obesity, and autoimmune diseases treatments (142). Taken together, it is clear that there is crosstalk between miRNA, metabolic, and inflammatory pathways in macrophages, and through an improved understanding of these networks, the next generation of targeted therapies will be developed to help fight human disease.

## Author Contributions

MN contributed in conceptualization, writing, reviewing, and editing this article. RO'C contributed in conceptualization, writing, reviewing, editing, and supervising this article. All authors contributed to the article and approved the submitted version.

## Conflict of Interest

The authors declare that the research was conducted in the absence of any commercial or financial relationships that could be construed as a potential conflict of interest.

## Abbreviations

A20: negative regulator of NF-kB encoded by *TNFAIP3*
ABCA1: ATP-Binding Cassette Transporter A1
AKT, also known as PKB: or Protein Kinase B
AMPK: 5′ AMP-activated protein Kinase
AP-1: Activator Protein 1
ARG1: Arginase
BIK: BCL-2 Interacting Killer
CCR7: C-C Motif Chemokine Receptor 7
CD206: Cluster of Differentiation 206, also known as Mannose Receptor
CD301: Cluster of Differentiation 301, also known as CLEC10A
C/EBP-δ: CCAAT/Enhancer-Binding Protein delta
CPT1a: Carnitine Palmitoyltransferase 1a
DLL4: Delta-Like Ligand 4
FIH1: Factor Inhibiting Hif-1
HIF1α: Hypoxia inducible factor 1α
IRAK1: Interleukin-1 Receptor-Associated Kinase 1
IRF4: Interferon Regulatory Factor 4
KLF13: Kruppel-Like Factor 13
LDL: Low-density Lipopolysaccharide
LIN28a: Lin28 homolog A, suppressor of Let-7 and SDH
mTOR: Mammalian Target of Rapamycin
MTP18: Mitochondrial Protein 18
NF-κB: Nuclear factor kappa B
NLR: Nod-like receptor
NO: Nitric Oxide
NOS_2_: Nitric Oxide Synthase
NOTCH1: Signaling receptor protein in the NOTCH family
OXPHOS: Oxidative Phosphorylation
PAMP: Pathogen associated molecular pattern
PAK1: P21-Activated Kinase 1
PFK-M: Phosphofructokinase, Muscle isoform
PI3K: Phosphatidylinositol 3-Kinase
PMA: Phorbol Myristate Acetate, a monocyte-to-macrophage inducer
PPP: Pentose phosphate pathway
PRR: Pattern recognition receptor
ROCK2: Rho-dependent Kinase 2
SCD2: Stearoyl-CoA Desaturase-2
SHIP1: Src Homology-2 domain-containing Inositol 5-Phosphatase 1
SOCS1: Suppressor of Cytokine Signaling 1
SREBF1: Sterol Regulatory Element-Binding Factor 1
SREBF2: Sterol Regulatory Element-Binding Factor 2
TCA Cycle: Tricarboxylic acid cycle
TET2: TET Methylcytosine Dioxygenase 2
TGF-β: Transforming Growth Factor-β
TLR: Toll-like receptor
TNF-α: Tumor Necrosis Factor-alpha
TRAF6: TNF Receptor Associated Factor 6
UDP-GlcNAc: Uridine Diphosphate *N*-Acetylglucosamine.
